# Chapped Hands

**Published:** 1875-12

**Authors:** 


CHAPPED HANDS—A simple remedy is found in every one's kitchen closet, and it is nothing more than common starch. Reduce it to an impalpable powder, put in a muslin bag, keep it in the table drawer. Whenever you take your hands out of dish-water or suds, wipe them dry with a soft towel, and while yet damp, shake the starch bag over them, and rub it in. The effect is most agreeable.

